# Potential Biological and Ecological Effects of Flickering Artificial Light

**DOI:** 10.1371/journal.pone.0098631

**Published:** 2014-05-29

**Authors:** Richard Inger, Jonathan Bennie, Thomas W. Davies, Kevin J. Gaston

**Affiliations:** Environment and Sustainability Institute, University of Exeter, Penryn, Cornwall, United Kingdom; Southern Illinois University, United States of America

## Abstract

Organisms have evolved under stable natural lighting regimes, employing cues from these to govern key ecological processes. However, the extent and density of artificial lighting within the environment has increased recently, causing widespread alteration of these regimes. Indeed, night-time electric lighting is known significantly to disrupt phenology, behaviour, and reproductive success, and thence community composition and ecosystem functioning. Until now, most attention has focussed on effects of the occurrence, timing, and spectral composition of artificial lighting. Little considered is that many types of lamp do not produce a constant stream of light but a series of pulses. This flickering light has been shown to have detrimental effects in humans and other species. Whether a species is likely to be affected will largely be determined by its visual temporal resolution, measured as the critical fusion frequency. That is the frequency at which a series of light pulses are perceived as a constant stream. Here we use the largest collation to date of critical fusion frequencies, across a broad range of taxa, to demonstrate that a significant proportion of species can detect such flicker in widely used lamps. Flickering artificial light thus has marked potential to produce ecological effects that have not previously been considered.

## Introduction

For many animals perception of light reflected from objects is the primary method used to gain information about their environment, and is critical for finding food and mates and for avoiding predation. A species' visual system must therefore be well adapted to its habitat and activity patterns in terms of spectral sensitivity, detectable light levels and the temporal resolution at which it can sample its environment. It has been known for some time that an evolutionary trade off between light sensitivity and temporal resolution exists, such that photoreceptors capable of detecting low light levels cannot sample the environment as frequently as can those operating at higher light intensities. Indeed, this trade-off was first recognised by Autrum [1], who demonstrated how the eyes of fast moving diurnal insects such as honey bees and dragonflies have much higher temporal resolution than do those of slow moving nocturnal species such as crickets and stick insects. This is also true for higher vertebrates, with, for example, fish living in clear waters with high light levels tending to have higher temporal visual resolution than do those found in turbid waters [2], and diurnal sharks found in shallower waters having higher temporal resolution than species found in deeper waters [3].

Animal visual systems, including their temporal resolution capabilities, have evolved across vast geological timescales with stable light regimes, provided almost exclusively by variation in sunlight (including that reflected by the moon). It is therefore unsurprising that there has been growing concern over the environmental implications of increasing artificial light levels, which typically differ significantly from natural light, and which have characteristics not necessarily optimal for highly adapted animal eyes. These differences have been shown to have implications for animal behaviour [4], [5], reproduction and mortality [6], [7], and community composition [8]. Such studies have, however, concentrated almost exclusively on the biological effects of the intensity, duration and, to a lesser extent, spectral signature of artificial light [9]. Another characteristic of many forms of artificial light has largely been overlooked. This is that the outputs of electrical lamps often flicker. The majority of modern artificial light sources connected to an alternating current (AC) will fluctuate or flicker, although the frequency and intensity of flicker will vary with the lighting technology. Incandescent bulbs flicker at the frequency of the electrical supply (50–60 Hz), although the intensity of flicker is low (low flicker index), whereas fluorescent lighting extinguishes and returns to full brightness twice over each voltage cycle (100–120 Hz), leading to a pronounced flicker effect (high flicker index). Light emitting diode (LED) technology is also increasing in popularity particularly for street lighting and these lamps also have a high flicker index. Indeed the perceived brightness of many LED lamps is controlled by regulating the flicker frequency.

The potential for widespread biological implications of flicker as a characteristic of artificial nighttime light is suggested by studies of the effects on humans. Major concerns have been raised about the impacts on human health and wellbeing of living and working under artificial lights, both for typical behavioural patterns in developed countries (e.g. regular periods of evening/night spent under artificial light), and for more acute exposures (e.g. night-shift workers). Documented effects of flicker include headaches, visual effects, and both neurological and physiological symptoms (Table 1). In some cases these arise under flicker rates that individuals can visually perceive. In others cases however they occur at higher rates which cannot be perceived by the individual but have been demonstrated to produce detectable physiological and neurological effects. In addition, there is some evidence, in a limited number of species, that flickering lamps can have a range of effects on non-human animals (Table 2) and that these effects can be produced by flicker that may or may not be perceivable by the animal.

**Table 1:** Documented effects of flickering artificial lights on humans.

Effects	Source of flicker	Frequency (Hz)	Reference
Headaches/Visual Effects	Low frequency fluorescent	100	[10]
Neurological Effects	Malfunctioning fluorescent	50	[11]
Neurological Effects	Amplitude-moderated flickering light	20–75	[12]
Neurological effects in photosensitive epileptics	Xenon gas discharge photo-stimulator	3–60	[13]
Physiological effects in agoraphobics	Low frequency fluorescent	100	[14], [15]
Seizures in photosensitive epileptics	Various	Various	[13]
Unperceived neurological effects	Light-emitting diode	Up to 200 Hz	[16]
Unperceived neurological effects	Computer monitor	42.5–75	[17]
Unperceived retinal effects	Various	76–162	[18]
Unperceived retinal effects	Cathode ray tube	76	[18]
Visual Effects	Low frequency fluorescent	100	[19]
Visual Effects	Cathode ray tube	50 & 100	[19]
Visual Effects	Low frequency fluorescent	120	[20]
Visual Effects	Computer monitor	70–110	[21]

**Table 2:** Documented effects of flickering artificial lights on animals.

Species common name	Species scientific name	Effects	Reference
Honeybee	*Apis mellifera*	Behavioural	[22]
Minute Pirate Bug	*Orius tristicolor*	Behavioural	[23]
White Fly	*Aleyrodidae*	Behavioural	[24]
Southern House Mosquito	*Culex quinquefasciatus*	Behavioural	[25]
Housefly	*Musca domestica*	Behavioural	[25]
Pink boll worm	*Pectinophora gossypiela*	Behavioural	[25]
House cricket	*Acheta domesticcus*	Behavioural	[25]
Housefly	*Musca domestica*	Behavioural	[26]
European Starling	*Sturnus vulgaris*	Physiological	[27]
European Starling	*Sturnus vulgaris*	Behavioural	[28]
European Starling	*Sturnus vulgaris*	Possible Physiological stress	[29]
European Starling	*Sturnus vulgaris*	Physiological stress & behavioral	[27]
European Starling	*Sturnus vulgaris*	Behavioural	[30]
European Starling	*Sturnus vulgaris*	Physiological stress & behavioral	[31]
Albino Rat	*Rattus norvegicus*	Physiological stress	[32]
Laboratory Mouse	*Mus musculus*	Visual	[33]

A key measure of the temporal resolution of vision systems is the critical fusion frequency (CFF), the threshold at which an animal ceases to perceive a flickering light source as a series of flashes, but rather as a continuous stream of light. An obvious first step towards assessing the likely effects of the flickering of artificial lights on animals other than humans is to determine in which species the CFF is higher than the flicker rates of widely used electrical lamps, and hence those that will perceive the light source as flickering. In addition, we explore whether the perception of flicker is segregated taxonomically or towards animals adapted to light, dark or variable natural light environments. Here we do this, building on the extensive, but scattered, literature in which CFF values have been reported.

## Materials and Methods

A literature search was conducted using ISI Web of Science, Google Scholar and Google. The ISI Web of Science search used the terms “flicker fusion frequency” and “critical flicker fusion” as topic fields spanning all years with no filters. The same terms were entered into Google Scholar and Google with no filters. Searches were conducted in September 2012. The Web of Science search produced 577 and 240 results for each term respectively. The Google Scholar search produced 4810 and 6150 results respectively whilst the Google search produced 23300 and 26400 results respectively. All Web of Science results were scanned for the use of non-human animals in the title and the resulting papers were downloaded from the University of Exeter electronic library for subsequent analysis. The first 1000 results for both search terms from Google Scholar and Google were also scanned for papers containing non-human animals in the title and these papers were then obtained either directly from the Internet or via the University of Exeter electronic library. All the resulting papers were then searched for data on the measurements of CFFs, which, when present, where added to the dataset along with the species name. Where CFFs were measured over a range of light intensities we took the mean measured value (CFF_mean_) under photopic conditions for use in the subsequent analysis. Where data were only presented graphically we extracted data from papers using Graph Click (Arizona Software). We also searched all papers for references to other papers reporting CFFs for non-human animals. Only data from peer-reviewed papers were used in the subsequent analysis. We also only included data from papers containing a full description of the methodology involved. CFF values are determined either by using electroretinography (ERG) to measure the electrophysiological response of the retina to flickering light of various frequencies, or by examining the spontaneous or taught behavioural response to flickering light. Using either method the CFF is taken as the frequency at which the subject ceases to respond to an increase in flicker frequency [34]. As behavioural methods often produce lower values than those of ERG, the method used to calculate CFF was included as a fixed factor in the analysis. In addition as different light sources with differing spectra characteristics may affect CFF values above and beyond the intrinsic CFFs of the species being measured we including light source (Incandescent, luminescent, gas discharge or monochromator) used to make the measurement within the analysis as a fixed factor.

It has been known for some time that animals adapted for low light environments tend to have lower CFFs than animals found in more intense light environments [3]. Hence we also included within the analysis a measure of the light intensity each species is likely to experience in natural situations. All diurnal animals were considered to be exposed to high light levels. Crepuscular and cathemeral animals were considered to be exposed to a variable light regime. Nocturnal and aquatic species inhabiting deep waters were considered as being exposed to low light levels.

To explore differences in CFF between taxonomic classes, natural lighting regimes and CCF calculation method we used linear mixed effects models (Gaussian error structure) fitted with the R (v3.0.2) language and environment [35], using the package ‘lme4’ [36]. Within the models CFF_mean_ was the dependent variable, taxonomic class, light level exposure, light source type and CCF calculation method were fixed factors, and species was a random factor (intercept only) to account for multiple data for some species. As the purpose of this study was only to identify groups of animals most likely to be affected by flicker we did not include a phylogenetic component to the model. To evaluate the variance explained by the model we calculated *R^2^_GLMM(m)_*, the marginal *R^2^* which describes the variance explained by the fixed factors and *R^2^_GLMM(c)_*, the conditional *R^2^* which is concerned with the variance explained by both the fixed and random factors [37]. Additional metrics of model fit were also calculated, these being the pseudo *R^2^_(N)_* of the models using the methods of Nagelkerke [38] and the % deviance explained by the fixed effects component of the model. This was achieved by comparing the deviance to a null (intercept only) model such that *% deviance = (deviance_null_–deviance_model_)/deviance_null_*. Package ‘MuMIn’ [39] was used for *R^2^* calculations. Package ‘lmerTest’ [40] was used to calculate F and p values using Satterthwaite [41] approximations to determine denominator degrees of freedom.

## Results

**Figure 1:**
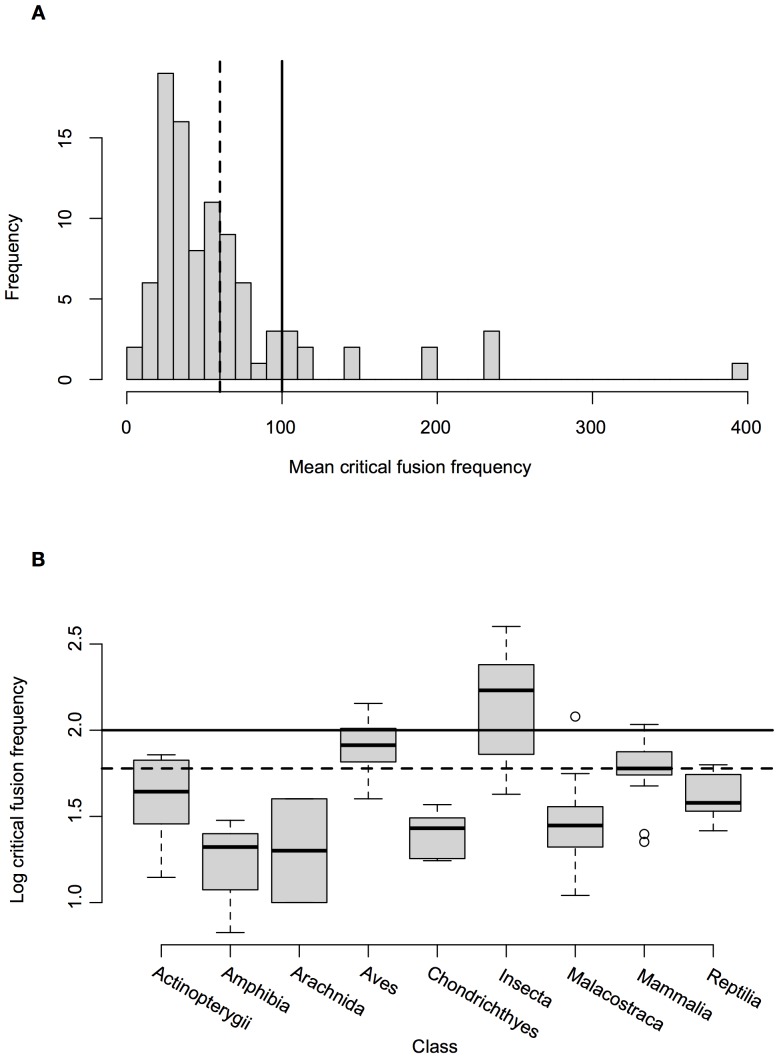
Distribution of critical fusion frequencies. (**a**) Histogram of the mean critical fusion frequency for all taxa; and (b) Boxplot of log_10_ critical fusion frequency (CFF) by class. The solid line indicates the flicker frequency of lamps on a 50 Hz electrical supply. The dotted line indicates mean CFF for humans. CFFs for Insecta are significantly higher and Amphibia significantly lower than for other classes.

We located 56 studies containing 93 measurements (19 behavioural & 74 using ERG) of CFF_mean_ for 81 species from 66 genera and 9 classes (Table 3, Table S1). We found a considerable range in CFF_mean_ values amongst species, with the lowest value of 6.7 Hz being obtained for the cane toad (*Bufo marinus*), and the highest value of 400 Hz for the black fire beetle (*Melanophila acuminate*) (Fig. 1A). In a considerable number of cases (16.1% of measurements, 13.5% of species, 16.6% of genera and 44.4% of classes), CFF_mean_ values were above the flicker frequency of fluorescent lamps on a 50 Hz electrical supply; i.e. 100 Hz. It should be noted that a number of countries utilise a 60 Hz electrical supply and hence the flicker frequency with be higher at 120 Hz.

**Table 3:** Species for which critical fusion frequencies have been measured.

Species common name	Species scientific name	Genus	Class	Light levels	Method	Mean CFF	Reference
Cane toad	*Bufo marinus*	Bufo	Amphibia	Low	ERG	6.7	[42]
Wolf spider	*Lycosa baltimoriana*	Lycosa	Arachnida	Low	ERG	10.0	[43]
Isopod	*Glyptonotus antarcticus*	Glyptonoyus	Malacostraca	Low	ERG	11.0	[44]
European eel	*Anguilla anguilla*	Anguilla	Actinopterygii	Low	Behavioural	14.0	[45]
Pandalid shrimp	*Plesionika rossignoli*	Plesionika	Malacostraca	Low	ERG	14.0	[46]
Little skate	*Leucoraja erinacea*	Leucoraja	Chondrichthyes	Low	ERG	17.5	[47]
Oplophorid shrimp	*Acanthephyra purpurea*	Acanthephyra	Malacostraca	Low	ERG	18.0	[46]
Blacknosed shark	*Carcharhinus acronotus*	Carcharhinus	Chondrichthyes	Variable	ERG	18.0	[3]
Penaeid shrimp	*Funchalia villosa*	Funchalia	Malacostraca	Low	ERG	21.0	[45]
Glass shrimp	*Pasiphaea multidentata*	Pasiphaea	Malacostraca	Low	ERG	21.0	[46]
Green frog	*Rana clamitans*	Rana	Amphibia	Low	ERG	21.0	[48]
Sergestid shrimp	*Sergestes arcticus*	Sergestes	Malacostraca	Low	ERG	21.0	[46]
Oplophorid shrimp	*Systellaspis debilis*	Systellaspis	Malacostraca	Low	ERG	21.0	[49]
Oplophorid shrimp	*Systellaspis debilis*	Systellaspis	Malacostraca	Low	ERG	21.0	[45]
Swordfish	*Xiphias gladius*	Xiphias	Actinopterygii	High	ERG	22.0	[50]
Harp seal	*Pagophilus groenlandicus*	Pagophilus	Mammalia	High	Behavioural	22.5	[51]
Oplophorid shrimp	*Janicella spinicauda*	Janicella	Malacostraca	Low	ERG	23.0	[46]
Euphausiid shrimp	*Meganyctiphanes norvegica*	Meganyctiphanes	Malacostraca	Low	ERG	23.0	[45]
Euphausiid shrimp	*Euphausia gibboides*	Euphausia	Malacostraca	Low	ERG	24.0	[46]
Penaeid shrimp	*Funchalia villosa*	Funchalia	Malacostraca	Low	ERG	24.0	[49]
Brown rat	*Rattus norvegicus*	Rattus	Mammalia	Low	ERG	25.0	[52]
Sergestid shrimp	*Sergia filictum*	Sergia	Malacostraca	Low	ERG	25.0	[49]
Anole lizard	*Anolis neckeri*	Anolis	Reptilia	High	ERG	26.1	[53]
Hammerhead shark	*Sphyrna lewini*	Sphyrna	Chondrichthyes	Variable	ERG	27.0	[3]
Euphausiid shrimp	*Nematoscelis megalops*	Nematoscelis	Malacostraca	Low	ERG	28.0	[46]
Anole lizard	*Anolis limifrons*	Anolis	Reptilia	High	ERG	28.5	[53]
Tiger salamander	*Ambystoma tigrinum*	Ambystoma	Amphibia	High	ERG	30.0	[54]
Oplophorid shrimp	*Janicella spinacaud*	Janicella	Malacostraca	Low	ERG	31.0	[49]
Bonnethead shark	*Sphyrna tiburo*	Sphyrna	Chondrichthyes	Variable	ERG	31.0	[3]
Oplophorid shrimp	*Janicella spinacauda*	Jancella	Malacostraca	Low	ERG	32.0	[49]
Oplophorid shrimp	*Oplophorus gracilirostris*	Oplophorus	Malacostraca	Low	ERG	32.0	[49]
Euphausiid shrimp	*Nematobrachion boopis*	Nematobrachion	Malacostraca	Low	ERG	33.0	[46]
Anole lizard	*Anolis valencienni*	Anolis	Reptilia	High	ERG	33.4	[53]
Euphausiid shrimp	*Stylocheiron maximum*	Stylocheiron	Malacostraca	Low	ERG	34.0	[46]
Anole lizard	*Anolis sagrei*	Anolis	Reptilia	High	ERG	34.4	[53]
Anole lizard	*Anolis carolinensis*	Anolis	Reptilia	High	ERG	34.6	[53]
Anole lizard	*Anolis grahami*	Anolis	Reptilia	High	ERG	34.7	[53]
Euphausiid shrimp	*Nematobrachion sexspinosus*	Nematobrachion	Malacostraca	Low	ERG	36.0	[46]
Euphausiid shrimp	*Stylocheiron maximus*	Stylocheiron	Malacostraca	Low	ERG	36.0	[49]
Lemon shark	*Negaprion brevirostris*	Negaprion	Chondrichthyes	Variable	ERG	37.0	[55]
Japanese rice fish	*Oryzias latipes*	Oryzias	Actinopterygii	Variable	ERG	37.2	[56]
Great horned owl	*Bubo virginianus*	Bubo	Aves	Low	ERG	40.0	[57]
Jumping spider	*Maevia inclemens*	Maevia	Arachnida	High	ERG	40.0	[58]
Anole lizard	*Anolis auratus*	Anolis	Reptilia	High	ERG	41.5	[53]
American cockroach	*Periplaneta americana*	Priplaneta	Insecta	Low	ERG	42.5	[59]
Green swordtail	*Xiphophorus helleri*	Xiphophorus	Actinopterygii	Variable	ERG	43.0	[60]
Euphausiid shrimp	*Nematobranchion flexipes*	Nematobranchion	Malacostraca	Low	ERG	44.0	[49]
Siamese fighting fish	*Betta splendens*	Betta	Actinopterygii	Variable	Behavioural	45.1	[61]
Domestic cat	*Felis domesticus*	Felis	Mammalia	High	ERG	47.5	[62]
Anole lizard	*Anolis gundlachi*	Anolis	Reptilia	High	ERG	50.0	[63]
Little owl	*Athene noctua*	Athene	Aves	Low	ERG	50.0	[64]
American crayfish	*Cambarus spp*	Cambarus	Malacostraca	Low	ERG	53.0	[65]
Hermit crabs	*Pagurus spp*	Pagursu	Malacostraca	Variable	ERG	53.0	[65]
Human	*Homo sapiens*	Homo	Mammalia	High	ERG	55.0	[66]
Decapod	*Jasus edwardsii*	Jasus	Malacostraca	Low	ERG	55.0	[67]
Tuatara	*Sphenodon punctatus*	Spenodon	Reptilia	Low	Behavioural	55.4	[68]
Tuatara	*Sphenodon punctatus*	Spenodon	Reptilia	Low	Behavioural	55.4	[69]
Euphausiid shrimp	*Nematobranchion sexspinosus*	Nematobranchion	Malacostraca	Low	ERG	56.0	[49]
Anole	*Anolis cristatellus*	Anolis	Reptilia	High	ERG	58.5	[63]
Human	*Homo sapiens*	Homo	Mammalia	High	Behavioural	60.0	[70]
Human	*Homo sapiens*	Homo	Mammalia	High	Behavioural	60.0	[71]
Tree shrew	*Tupaia belangeri*	Tupaia	Mammalia	High	Behavioural	60.0	[72]
Rhesus macaque	*Macaca mulatta*	Macaca	Mammalia	High	Behavioural	61.0	[73]
Anole lizard	*Anolis pulchellus*	Anolis	Reptilia	High	ERG	63.0	[63]
Domestic chicken	*Gallus domesticus*	Gallus	Aves	High	Behavioural	63.5	[74]
Migratory locust	*Locusta migratoria*	Locusta	Insecta	High	ERG	65.0	[59]
American red squirrel	*Tamiasciurus hudsonicus*	Tamiasciurus	Mammalia	High	ERG	65.0	[75]
Threespined stickleback	*Gasterosteus aculeatus*	Gasterosteus	Actinopterygii	Variable	Behavioural	67.0	[76]
Guppy	*Poecilia reticulata*	Poecilia	Actinopterygii	High	Behavioural	67.0	[76]
Short-eared owl	*Asio fammeus*	Asio	Aves	Variable	ERG	67.5	[77]
Chinese tussah moth	*Antheraea pernyi*	Antheraea	Insecta	Low	ERG	70.0	[59]
Domestic chicken	*Gallus domesticus*	Gallus	Aves	High	Behavioural	71.5	[78]
Salmon	*Salmo salar*	Salmo	Actinopterygii	Variable	ERG	72.0	[79]
Domestic dog	*Canis familiaris*	Canis	Mammalia	High	Behavioural	75.0	[80]
Emperor moth	*Saturnia pavonia*	Saturnia	Insecta	Low	ERG	75.0	[59]
Rock Pigeon	*Columba livia*	Columba	Aves	High	Behavioural	77.0	[81]
Fruit fly	*Drosophila hydei*	Drosophila	Insecta	High	ERG	80.0	[59]
Domestic Chicken	*Gallus domesticus*	Gallus	Aves	High	Behavioural	87.0	[82]
Rhesus Monkey	*Macaca mulatta*	Macaca	Mammalia	High	ERG	95.0	[83]
Rock Pigeon	*Columba livia*	Columba	Aves	High	ERG	100.0	[77]
Starling *	*Sturnus vulgaris*	Sturnus	Aves	High	ERG	100.0	[84]
Domestic Chicken	*Gallus domesticus*	Gallus	Aves	High	ERG	104.0	[85]
Domestic Chicken	*Gallus domesticus*	Gallus	Aves	High	Behavioural	105.0	[86]
Yellow-pine Chipmunk	*Neotamias amoenus*	Neotamis	Mammalia	High	ERG	108.0	[75]
Ground squirrel	*Spermophilus laterali*	Citellus	Mammalia	High	ERG	108.0	[75]
Rock louse	*Ligia occidentalis*	Ligia	Malacostraca	High	ERG	120.0	[87]
Rock Pigeon	*Columba livia*	Columba	Aves	High	ERG	143.0	[88]
Tsetse fly	*Glossina morsitans*	Glossina	Insecta	High	ERG	145.0	[59]
Honeybee	*Apis mellifera*	Apis	Insecta	High	Behavioural	200.0	[89]
Honeybee	*Apis mellifera*	Apis	Insecta	High	Behavioural	200.0	[90]
Dragonflies	*Anisoptera*	Anisoptera	Insecta	High	ERG	240.0	[91]
Honeybee	*Apis mellifera*	Apis	Insecta	High	ERG	240.0	[92]
Blow-fly	*Calliphoridae*	Calliphora	Insecta	High	ERG	240.0	[1]
Black Fire Beetle	*Melanophila acuminata*	Melanophila	Insecta	High	ERG	400.0	[93]

*Indirect evidence suggesting CFF<100 Hz.

The mixed effects model explained a substantial proportion of the variation in CFF_mean_ (*R^2^_GLMM(m)_* = 0.70, *R^2^_GLMM(c)_* = 0.94, *R^2^_(N)_* = 0.76, *% deviance*  = 0.55) and highlighted significant differences between the taxonomic classes (F_[8,81]_ = 9.47, p<0.0001; Fig 1B) and between levels of light exposure (F_[2,80]_ = 17.63, p<0.0001) but no differences were found between the methods used to calculate CFF (F_[2,22]_ = 1.10, p = 0.30) and the lighting source used (F_[2,40]_ = 0.54, p = 0.70, data were available for 71 of the 93 studies). To further ensure that these differences were not related to the methodology used to obtain the CFF measurements we conducted the analysis on a subset of data where CFF values had been measured only by ERG, which retained significant differences between taxonomic classes (F_[8,71]_ = 14.37, p<0.0001) and levels of light exposure (F_[2,71]_ = 25.15, p<0.0001).

Nocturnal animals had the lowest CFF values (mean  = 32.09, SD = 16.73) compared to animals exposed to variable lighting regimes (mean  = 45.25, SD = 17.77) and diurnal animals (mean  = 92.21, SD = 75.72), which had significantly higher CFF values.

The lowest CFF values were found within the amphibians (mean = 19.23, SD = 11.75), followed by arachnids (mean = 25,SD = 21.21), Chondrichthyes (mean  = 26.1, SD = 8.41), and malacostracans (mean  = 33.7,SD = 21.26), although one intertidal species, the tock louse (*Ligia occidentalis*), had a CFF_mean_ of 120 Hz. All the reptiles (mean  = 42.94, SD = 12.79) and actinopterygians (mean  = 45.9,SD = 21.53) had CFF values below 100 Hz, as did most mammals (mean  = 64.77, SD = 26.77) with the exception of two species, the yellow-pine chipmunk (*Neotamias amoenus*) and the golden-mantled ground squirrel (*Spermophilus laterali*) which both had CFF_mean_ values of 108 Hz. Birds (mean  = 82.23, SD = 28.97) had the second highest CFF_mean_, although two of the six species of birds within the dataset were nocturnal owls. When the diurnal species were considered independently CFF_mean_ rose to 93.38, with domestic chickens (*Gallus domesticus*) (one out of five measurements), pigeons (*Columba livia*) (two out of three measurements), and the common starling (*Sturnus vulgaris*) all having CFF_mean_ values above 100 Hz. Insects (mean  = 166.46, SD = 106.29) had by far the highest CFF_mean_ values, although there was a distinct difference between nocturnal (mean  = 70) and diurnal species (mean  = 201.1), a pattern first noted by Autrum [1]. Most diurnal insects, including dragonflies Anisoptera spp., honeybees (*Apis mellifera*), blow-flies *Calliphoridae* spp., tsetse flies (*Glossina morsitans*) and black fire beetles had CFF values greater than 100 Hz, apart from the fruit fly (*Drosophila* hyde)i and migratory locust (*Locusta migratoria*).

A subset of the dataset of those species with CFFs of over 100 Hz also measured CFFs over a range of light intensities, allowing us to estimate the minimal light intensity required to produce such values. Tsetse flies produced a CFF of 286 at 300 lux, which dropped to 101 Hz at 3 lux. For domestic chickens, CFFs of greater than 100 Hz can be achieved at around 250 cd/m^2^ (approximately 150 lux) [85]. Srinivasan & Lehrer [89] measured CFF for honeybees at two light intensities, 3000 lux, producing a CFF of 200 Hz, and 300 lux, producing a value of 144 Hz. Interpolating from these data we predict a light level of around 138 lux would be needed to produce a CFF of 100 Hz.

## Discussion

Our results clearly demonstrate how a significant proportion of animals, particularly fast moving diurnal birds and insects have the potential to perceive the flicker of electric lamps, which has been demonstrated to have detrimental effects on both human and non-human species. When we also consider that in the case of humans, flicker can produce symptoms when it cannot be perceived (but can invoke measurable physiological changes [16], [17], [18]) we suggest that, in addition, any species with a CFF of 60 Hz (as in humans) or higher, including many other mammals, and some crustaceans, reptiles and fish, have the potential to be affected by flicker.

This said, the perception of flicker is likely to be limited in many natural situations for a number of reasons. First, all animals able to detect artificial flicker are naturally diurnal hence only species for which artificial light serves effectively to increase day length, or which are facultatively nocturnal, are likely to be affected [9]. Second, CFF decreases with light intensity, which itself decays with distance from the light source. Hence lamps need to produce light that, once it reaches the animal, is still sufficiently intense to induce CFFs that are high enough for animals to perceive the flicker. For example, CFFs in Tsetse flies remain relatively high at low light intensities, such that individuals will still be able to perceive flickering street lamps at 3 lux, which is approximately the lowest intensity light found at ground level between neighbouring metal halide lamps [94]. Whereas in honeybees and domestic chickens a much greater light intensity is required, such that, based on the output from a typical metal halide street lamp (Phillips Cosmopolis 80 W, measured by JB), a subject would need to be closer than three metres to the light source in order to perceive the flicker.

The evidence presented here suggests that impacts from the perception of the flicker of artificial light may be rather limited for most species studied thus far. There remains, however, a scarcity of data on CFFs for many taxa that are most likely to be affected, hence we suggest that the potential ecological effects should not be wholly dismissed. Considering the case of insects, CFFs have only been characterised for nine diurnal and three nocturnal species, producing a wide range of values (42.5–400 Hz). Given the vital role insects play in ecosystem functioning (e.g. pollination, decomposition), and as nutrition for other organisms, this depauperate knowledge base should give cause for concern. Those species for which we have data suggest that a wide range of insects have the potential to be affected by flicker and as such should be a key target for further research. To the best of our knowledge, quantification of CFFs has only been carried out in five species of birds (although CFFs of greater than 100 Hz have been inferred for starlings), with considerable variation within the range of values being reported (40–143 Hz). More importantly, we only have direct measurement of CFFs for two diurnal species. One of these is the domestic chicken, whose wild counterparts are weakly flying, mostly ground dwelling birds, adapted to living under the forest canopy which are unlikely to move rapidly enough to have adapted a particularly high temporal sensitivity. The only other diurnal species of bird for which CFFs have been quantified is the pigeon, a very common urban bird that is likely to encounter high levels of artificial light. Pigeons have the highest CFF values recorded (143 Hz) of any vertebrate species and will easily be able to perceive flicker from artificial lights. From an evolutionary perspective, it is likely that other birds capable of rapid flight have high CFFs, and are able to detect flickering artificial light.

**Figure 2:**
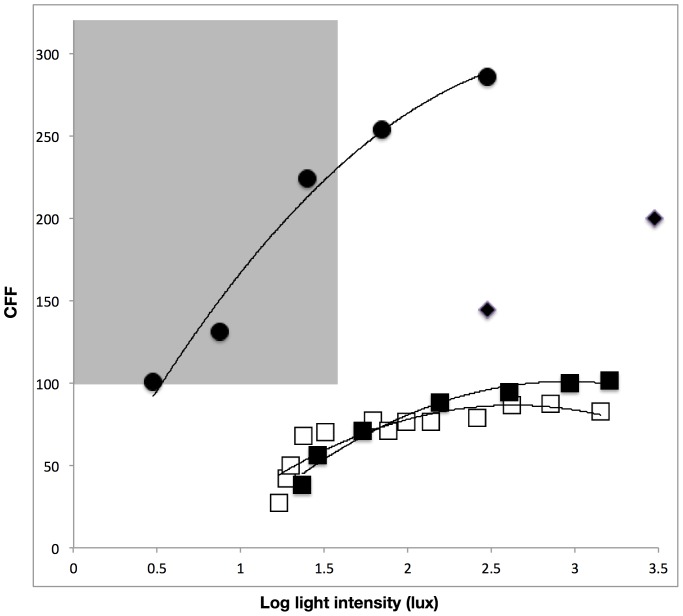
CFF responses to light intensity. How CFF decreases with light intensities for different species. Squares indicate data collected from domestic chickens, with empty squares being from behavioural measurements (y = −21.5x^2^+113.1x−62.6, R^2^ = 0.8, data from Linsey et al. 2011) and filled squares from ERG measurements (y = −22.4x^2^+132.2x−94.1, R^2^ = 0.97, data from [82]). Solid circles indicate data collected from tsetse flies (y = −30.6x^2^+118.4x−8.9, R^2^ = 0.98, data from [59]). Two data points were available for honey bees, taken from [89]; shown as filled diamonds). Shaded area highlights the parameter space within which flicker from 50 Hz supply lamps will be perceptible under light intensities commonly produced by artificial lamps within the environments (0–40 lux [9], [94]).

For both birds and insects there is substantial variation in the range of CFFs recorded. A component of this variation may be explained by the method by which CFF measurements were made, as behavioural mechanisms are generally considered to produce lower values than those obtained via ERG, because of post-retina neurological processing [34], though this study found no effect of experimental method. There remains however, considerable variation within measurements for each method, suggesting other causes. Understanding of what drives variation in temporal sensitivity within and between taxa remains poor, beyond the fact that CFFs are related to the light levels to which animals are exposed and tend to scale with metabolic rate and body size [95]. At the level of the individual a number of factors have been found to influence CFF, most importantly light intensity, with CFF increasing linearly with the logarithm of light intensity (within certain limits) for humans [96], [97]. The shape of this relationship and how it varies within and between taxa are key to understanding the potential impacts of flicker from artificial light, but have only been determined for a limited number of non-human species [82]. For example, tsetse flies will be able to perceive flicker within a range of light intensities likely to be produced by artificial lighting sources within the environment (Fig. 2), where as for domestic chickens their ability to perceive flicker is at the very limits of their visual capabilities. As such chickens will only be able to perceive flicker at light intensities exceeding the range likely to be produced by artificial lamps.

Our results suggest that diurnal species are most likely to be affected by flicker as nocturnal species studied up until now have CFFs too low to perceive flicker, and many nocturnal species tend to shun artificially lit areas. There remains however a number of nocturnal animals groups likely to be affected by flickering lights, these being animals which move rapidly in light environments. This presents a particular challenge for visual systems, as generally there is a trade-off between detectable light levels and the temporal resolution at which they can sample the environment. Potential groups prone to being affected by flicker include, but are not limited to, nocturnal flying insects, for example hawkmoths, nocturnal flying birds (including some Charadriiformes, Caprimulgiformes and Strigiformes) and bats. Whilst bats primary mechanism of navigation is via echolocation, there exists increasing evidence that many species rely to a varying extent on vision [98]. In addition, it is becoming increasingly apparent that some species of bats often utilise artificially lit areas as they act to concentrate their food resources, which often aggregate around the lamps [99], [100]. Given these factors it seems prudent further to investigate the visual systems of these groups in order to identify those with the potential to be affected by the flickering of artificial lamps.

Explicit studies of the biological effects of light flicker on animals remain scarce, and have focused on the efficacy of trapping devices for insects and on welfare issues for captive vertebrates. Nonetheless, those that have been conducted provide further support for a potentially widespread impact (Table 2), as flicker has been found to influence behavioural and movement patterns, visual systems and levels of stress.

A wide range of taxa are likely to be able to detect flicker, and it has been found to produce detrimental effects in both human and non-human animals. We suggest that flickering from electric lamps represents a potential environmental impact from artificial light pollution which has not previously been considered. Given the precautionary principle and that it is likely that in the future an increasing level of artificial light will come from lamps with a high flicker index, as incandescent bulbs are phased out, and replaced with fluorescent (including compact fluorescent bulbs, generally referred to as ‘low energy bulbs’) and LED lamps, we suggest the impacts of flickering light on natural systems are given urgent attention.

## Supporting Information

Table S1 Species for which critical fusion frequencies have been measured. Includes the light source type used in the measurement of CFF values. Species are ordered by class.
(XLSX)
